# Management of hepatitis B virus prophylaxis in patients treated with disease-modifying therapies for multiple sclerosis: a multicentric Italian retrospective study

**DOI:** 10.1007/s00415-022-11009-x

**Published:** 2022-02-14

**Authors:** Antonio Riccardo Buonomo, Giulio Viceconte, Massimiliano Calabrese, Giovanna De Luca, Valentina Tomassini, Paola Cavalla, Giorgia Teresa Maniscalco, Diana Ferraro, Viviana Nociti, Marta Radaelli, Maria Chiara Buscarinu, Damiano Paolicelli, Alberto Gajofatto, Pietro Annovazzi, Federica Pinardi, Massimiliano Di Filippo, Cinzia Cordioli, Emanuela Zappulo, Riccardo Scotto, Ivan Gentile, Antonio Luca Spiezia, Martina Petruzzo, Marcello De Angelis, Vincenzo Brescia Morra, Claudio Solaro, Claudio Gasperini, Eleonora Cocco, Marcello Moccia, Roberta Lanzillo, Raising Italian Researchers in Multiple Sclerosis (RIREMS) study group

**Affiliations:** 1 Department of Clinical Medicine and Surgery—Section of Infectious Diseases, University of Naples “Federico II”, Via Sergio Pansini 5, 80130 Naples, Italy; 2 Department of Neuroscience, Biomedicine and Movement, The Multiple Sclerosis Center of the University Hospital of Verona, Verona, Italy; 3 Institute for Advanced Biomedical Technologies (ITAB), Department of Neurosciences, Imaging and Clinical Sciences, University “G. d’Annunzio” of Chieti-Pescara, Chieti, Italy; 4 Department of Neuroscience and Mental Health-City of Health and Science University, Hospital of Torino, Multiple Sclerosis Center, Turin, Italy; 5 Multiple Sclerosis Center, “A. Cardarelli” Hospital, Naples, Italy; 6 Department of Biomedical, Metabolic and Neurosciences, University of Modena and Reggio Emilia, Modena, Italy; 7 Institute of Neurology, Fondazione Policlinico Universitario ‘A. Gemelli’ IRCCS, Catholic University of Sacred Heart, Rome, Italy; 8 Department of Neurology and Multiple Sclerosis Center, ASST Papa Giovanni XXIII, Bergamo, Italy; 9 Department of Neuroscience, Mental Health and Sensory Organs, Sapienza University, S. Andrea Hospital Site, Rome, Italy; 10 Department of Basic Medical Sciences, Neuroscience and Sense Organs, University of Bari “Aldo Moro”, Bari, Italy; 11 Multiple Sclerosis Center, ASST Valle Olona–Gallarate Hospital, Gallarate, VA Italy; 12 UOSI Multiple Sclerosis Rehabilitation, IRCCS, Bologna, Italy; 13 Section of Neurology, Department of Medicine and Surgery, University of Perugia, Perugia, Italy; 14 Multiple Sclerosis Center, Montichiari Hospital, ASST Spedali Civili Di Brescia, Brescia, Italy; 15 Department of Neurosciences, Reproductive and Odontostomatological Sciences, University of Naples “Federico II”, Naples, Italy; 16 CRRF Mons. Luigi Novarese, Moncrivello, VC Italy; 17 Department of Neuroscience, San-Camillo Forlanini Hospital, Rome, Italy; 18 Multiple Sclerosis Center, Binaghi Hospital, Azienda Tutela Della Salute (ATS) Sardegna, Cagliari, Italy

**Keywords:** Multiple sclerosis, Hepatitis B, Ocrelizumab, Rituximab, Cladribine, Vaccination

## Abstract

**Background:**

Patients with multiple sclerosis (MS) often receive disease-modifying therapies (DMTs) that can expose them to reactivation of potential occult hepatitis B virus (HBV) infection (pOBI). We aimed to evaluate the MS Centers behavior regarding HBV screening and prophylaxis in a large cohort of MS patients receiving anti-CD20 or cladribine.

**Methods:**

Retrospective, multicentric study recruiting Italian MS patients treated with rituximab, ocrelizumab and cladribine.

**Results:**

We included 931 MS patients from 15 centers. All but 38 patients performed a complete HBV screening. Patients’ age > 50 years was significantly associated with no history of vaccination and HBsAb titres < 100 mIU at baseline (*p* < 0.001). No significant correlation was found between post-vaccination HBsAb titres and type of treatment (*p* = 0.5), pre-or post-therapy vaccination (*p* = 0.2) and number of previous DMTs (*p* = 0.2). Among pOBI patients (*n* = 53), 21 received antiviral prophylaxis, while only 13 had HBV DNA monitoring and 19 patients neither monitored HBV DNA nor received prophylaxis.

**Conclusions:**

Baseline HBV screening in patients receiving anti-CD20 and cladribine is a consolidated practice. Nonetheless, HBV vaccination coverage is still lacking in such population and age is a significant factor associated with low HBV protection. Rituximab, ocrelizumab and cladribine did not impair HBV vaccine response. Almost 35% of pOBI patients fail to receive HBVr prevention. Management of HBV prophylaxis could be improved in MS patients and further prospective studies are needed to assess the effectiveness of prophylactic strategies in such patients.

## Background

Multiple sclerosis (MS) is a demyelinating immune-mediated disease of the central nervous system with an estimated prevalence of 30 cases per 100 000 inhabitants globally and 176 cases per 100 000 inhabitants in Italy, representing the most common cause of non-traumatic disability in young adults [1, 2]. Disease-modifying therapies (DMTs) for MS have an immunomodulatory effect, reducing inflammation without depleting lymphocytes, or an immunosuppressive effect, as it occurs with preferential B-cells depleting nucleoside analogs (cladribine) or selective B-cell depleting monoclonal antibodies (rituximab, ocrelizumab) [3].

B-cell depleting therapies are associated with potential risks of viral infections. Hepatitis B virus (HBV) infection is the most common chronic viral infection, with estimated 30% of the world population having serological evidence of current or past infection [4]. Occult HBV infection (OBI) is defined as the presence of HBV DNA in the liver—with or without detectable serum HBV DNA—in the absence of detectable HBsAg. OBI is defined potential (pOBI) if HBV DNA in liver cannot be ruled out, but patient have HBcAb and/or anti-HBs detectable without HBsAg [5].

As such, B-cell depleting therapies potentially hold the risk of pOBI reactivation, also leading to acute liver failure [6]. Risk of reactivation is well known in patients treated with rituximab for hematology conditions, reaching 40% in HBsAg positive patients and ranging from 2 to 23% in pOBI patients [7]. Conversely, in other settings in which anti-CD20 are used, such as rheumatology, reactivation risk is estimated to be lower, since the underlining immunological asset is expected to be less compromised than haematologic conditions. For instance, in patients treated with rituximab for rheumatoid arthritis, the incidence of reactivation is estimated to be negligible and a baseline HBsAb titer > 100 has been demonstrated to be protective [8]. Nonetheless, fatal cases of HBV reactivation have been reported in patients with neurological autoimmune disorders treated with anti-CD20 [9, 10].

No definite evidence has been provided so far concerning the best strategy to prevent HBV reactivation among MS patients, although the European Society of Clinical Microbiology and Infectious Diseases recommends administering antiviral prophylaxis for HBsAg positive and pOBI patients that receive anti-CD20 therapies, regardless of the baseline condition [11]. There are no current guidelines specifically aimed at managing pOBI, but recommendations are derived from the international guidelines on general HBV management from the European Association for the Study of the Liver (EASL), and the American Association for the Study of Liver Diseases (AASLD) [6, 12]

As for cladribine, fewer data are available since HBsAg-positive patients have been excluded from phase III trials for its use in MS and no data about participants’ HBV serostatus have been reported, although anecdotal cases of HBV reactivation following cladribine administration for MS have been described [13].

Vaccination is the best strategy to prevent HBV infection. Although HBV vaccination is compulsory in Italy for all the newborns since 1991, according to the Italian viral hepatitis surveillance system (SEIEVA), the incidence of acute HBV infections in 2018 in Italy was still 0.4 cases/100 000 people; young adults (35–55 years) are the most represented group and 7.6% of new infections are reported among individuals who received vaccination, although in most cases with an incorrect schedule [14]. Notably, it is well established that DMTs for MS, especially anti-CD20, can reduce the response to several vaccinations, but no data are available on HBV seroprevalence and response to HBV vaccination in such population [15]. The fact that the highest incidence of acute HBV infection occurs in the same age group in which the incidence of MS is higher, compels to better investigate and expand vaccination coverage of this population [16].

### Study aims

We aimed to evaluate in a population of patients with MS and receiving ocrelizumab, rituximab or cladribine the following variables: (i) the proportion of MS patients who received complete HBV serological screening before commencing on ocrelizumab, rituximab or cladribine, defined as the availability in the clinical records of HBsAg, HBcAb-IgG and HBsAb before starting the DMT; (ii) the proportion of patients vaccinated for HBV before starting DMT therapy; (iii) the difference in pre- and post-vaccination HBsAb titers and its association with timing of vaccination, therapeutic history and age; (iv) choice of HBV reactivation prevention strategy (pre-emptive or antiviral prophylaxis) in pOBIs and its association with baseline HBsAb titers.

## Patients and methods

This is a multicentric, retrospective, cross-sectional study involving 15 MS Centers across Italy. We retrospective examined the clinical records of patients with MS who are currently treated with ocrelizumab, rituximab or cladribine.

Clinical files from participating studies were reviewed by each involved center from July 2020 to January 2021, with cases being included from 2017 onwards.

Inclusion criteria were patients older than 18 years, affected by any form of MS, currently receiving ocrelizumab, rituximab or cladribine or that have received at least one dose of ocrelizumab, rituximab or cladribine in the last 6 months.

Exclusion criteria were concurrent DMTs or high-dose steroids (> 20 mg/daily of prednisone or equivalent); patients that have received plasma-exchange during life or intravenous immunoglobulins in the last 6 months.

The following data have been collected: demographic and clinical characteristics; HBV baseline serostatus; previous medication history for MS; history of HBV vaccination during lifetime; history of HBV vaccination during follow-up at MS Center; HBsAb titres at baseline and after HBV vaccination.

Patient’s data were collected in an electronic database. Each MS Center was asked to send back the filled database. GV, ARB, MM, SR jointed and formatted the received data. No data were deleted from the original databases.

### Statistical analysis

Categorical variables were confronted using bivariate logistic regression analysis; continuous variables were confronted to continuous variables using Spearman’s Rho correlation and to categorical variables with Mann–Whitney *U* test for dichotomous variables or with Kruskal–Wallis test for categorical variables with more than 2 values. A significance level of 0.05 was set for the interpretation of the results. Statistical analyses were performed using IBM SPSS Statistics version 27.

## Results

### Study population

We included 931 MS patients (median age: 47, IQR 38–54 years; females: 559, 60%) treated with ocrelizumab (*n* = 738, 79.3%), rituximab (*n* = 69, 7.4%), or cladribine (*n* = 124, 13.3%) from 15 Italian MS Centers. Follow-up was available for 747 patients (15.9 ± 9.1 months). Demographics and clinical features are reported in Table 1.

**Table 1:** Demographics and clinical features of enrolled patients (*N* = 931)

	Ocrelizumab *N* = 738	Rituximab *N* = 69	Cladribine *N* = 124	Total *N* = 931
Age, years, median (IQR)	47 (39–55)	51 (42–59)	39 (29–47)	47 (38–54)
Female, *n* (%)	434 (58.8)	49 (71)	85 (68.5)	559 (60)
Treatment naïve, *n* (%)	182 (24.6)	26 (37.6)	31 (25)	239 (25)
Availability of follow-up, *(%)*	593 (80.3)	60 (86.9)	94 (75.8)	747 (80)
Follow-up duration, months ± SD	16.9 ± 8.9	20.0 ± 11.8	8.8 ± 5.8	15.9 ± 9.1
Previous DMT, *n* (%)				
*Alemtuzumab*	19 (2.6)	2 (2.9)	0 (0)	
*Azathioprine*	28 (3.8)	5 (7.2)	1 (0.8)	
*Cyclophosphamide*	14 (1.9)	2 (2.9)	0 (0)	
*Cladribine*	2 (0.3)	0 (0)	0 (0)	
*Daclizumab*	2 (0.3)	0 (0)	0 (0)	
*Dimethyl fumarate*	113 (15.3)	2 (2.9)	36 (29)	
*Fingolimod*	108 (14.6)	6 (8.7)	23 (18.5)	
*Glatiramer acetate*	51 (6.9)	2 (2.9)	7 (5.6)	
*Interferon beta*	51 (6.9)	6 (8.7)	16 (12.9)	
*Methotrexate*	3 (0.4)	2 (2.9)	0 (0)	
*Mitoxantrone*	1 (0.1)	4 (5.8)	0 (0)	
*Natalizumab*	74 (10)	5 (7.2)	7 (5.6)	
*Rituximab*	51 (6.9)	0 (0)	0 (0)	
*Siponimod*	4 (0.5)	0 (0)	0 (0)	
*Teriflunomide*	46 (6.2)	2 (2.9)	4 (3)	

*DMT* disease-modifying therapy

### HBV screening

All (*n* = 931) but 38 patients (4%) had completed HBV screening before starting treatment. Of 38 patients with incomplete screening, 24 were treated with ocrelizumab, 13 with rituximab and 11 with cladribine. Among patients who received a complete screening (*n* = 893), no patients resulted positive to HBsAg. However, 310 patients (34%) were positive to HBsAb, of which 41 (4%) also resulted in HBcAb-IgG positive, as from the previous infection.

### Vaccination history and pOBI

Among the 269 HBsAb-positive and HBcAb-IgG-negative patients, 134 (15%) reported previous vaccination, while 6 reported no history of vaccination and 129 had vaccination history missing; 7 patients resulted in HBcAb-IgG positive in the absence of HBsAb. Thus, 53 patients (6%) were considered as pOBI (41 HBsAb + /HBcAb + ; 6 HBsAb + /HBcAb- not vaccinated; 6 HBsAb-/HBcAb +). Only 184 out of 310 HBsAb positive patients presented with tires > 100 mIU/mL (51.2%). Detailed results of HBV screening are reported in Table 2.

**Table 2:** HBV screening at baseline

Patients with complete baseline screening	Ocrelizumab *N* = 714	Rituximab *N* = 56	Cladribine *N* = 113	Total *N* = 893
HBsAg, positive (%)	0 (0)	0 (0)	0 (0)	0 (0)
HBsAb positive—HBcAb negative, *n* (%)	209 (29)	11 (19)	49 (43)	269 (30)
Previous infection, *n* (%)	33 (5)	7 (12)	1 (0.8)	41 (4)
HBV vaccination	112 (15)	2 (3)	20 (17)	134 (15)
HBsAb > 100 mIU/mL, *n* (%)	154 (21)	6 (1)	24 (21)	184 (20)
pOBI, *n* (%)	38 (5)	9 (16)	6 (5)	53 (6)

Previous infection: HBsAb-positive, HBcAb-IgG-positive patients; *pOBI* potential occult HBV infection

### Vaccination response

Among 893 patients who received complete screening, quantitative HBsAb was available for 865 patients: among them, 681 presented with baseline HBsAb titer negative or < 100 mIU/mL. Age > 50 years was significantly associated with no history of vaccination and HBsAb titres < 100 mIU at baseline (*p* < 0.001), while number of previous DMTs > 2 (*p* = 0.8) was not (Table 3).

**Table 3:** HBV management strategies

Baseline HBsAb titres, *n* (%)	Patients with complete baseline screening *N* = 893				
	Negative or < 100 mIU/mL 681 (76)	> 100 mIU/mL 184 (20)	OR	95%CI	*P* value
Age, *n* (%)					
≤ 50 years	406 (58)	134 (74)	2.01	1.4–2.9	< 0.001
> 50 years	291 (41)	45 (25)			
Number of previous DMTs, *n* (%)					
< 1	305 (47)	80 (46)			
> 1	349 (52)	88 (53)	1.04	0.74–1.46	0.8
Previous vaccination during lifetime, *n* (%)	Vaccination 134 (15)	No vaccination 509 (56)			
Age, *n* (%)					
≤ 50 years	116 (86)	242 (47)	7.1	4.2–12	< 0.001
> 50 years	18 (13)	267 (52)			
	pOBI patients *n* = 53 (5.9%)				
Baseline HBsAb titres, *n* (%)	Negative or < 100 mIU/mL 13 (24)	> 100 mIU/mL 40 (75)			
Antiviral prophylaxis	6 (46)	15 (37)	1.1	0.7–1.3	0.6
HBV DNA monitoring	0 (0)	13 (32)	1	0.67–1.48	0.1
No intervention	7 (53)	12 (30)	1.31	0.74–2.33	0.3
	Pre- or post-therapy vaccinated patients *n* = 86 (9.6%)				
Post-vaccination titres, mIU/mL, median (IQR)	61.5 (1.25–168.5)				
Number of previous DMTs, median (IQR)	2 (1–2)				0.2
Type of treatment					0.5
Ocrelizumab, *n* (%)	66 (76.7)				
Rituximab, *n* (%)	3 (3.4)				
Cladribine, *n* (%)	14 (1.5)				
Timing of vaccination					0.2
Pre-therapy	81 (94)				
Time to follow-up, months, mean ± SD	5.4 ± 3.6				
Post-vaccination titres, mIU/mL, median (IQR)	61.5 (1.25–191.5)				
Post-therapy	5 (5.8)				
Time to follow-up, months, mean ± SD	5.9 ± 3.2				
Post-vaccination titres, mIU/mL, median (IQR)	205 (2–205)				

*DMT* disease-modifying therapy

Globally, 81 patients received HBV vaccination before commencing on DMT (ocrelizumab = 64; rituximab = 3; cladribine = 14). Among them, only 15 (ocrelizumab = 13; rituximab = 1; cladribine = 1) had HBsAb titer available, which was performed after a mean of 5.4 ± 3.6 months after DMT’s administration, and the median titer was 61.5 mIU/mL (IQR 1.25–191.5).

In addition, 5 patients received HBV vaccination after commencing DMTs (ocrelizumab = 4; rituximab = 1); 4 of them had HBsAb titer checked after a mean time of 5.9 ± 3.2 months (ocrelizumab = 3; rituximab = 1), with a median titer of 205 mIU/mL (IQR 2–205), and only 1 patient treated with ocrelizumab remained below protective levels. No significant correlation has been found between post-vaccination HBsAb titres (*n* = 19) and type of treatment (*p* = 0.5); pre-or post-therapy vaccination (*p* = 0.2) and number of previous DMTs (*p* = 0.2).

### Prevention strategies

Among patients potentially at risk of HBV reactivation (pOBI, *n* = 53), 20 received antiviral prophylaxis with lamivudine and 1 with entecavir, while only 13 had HBV DNA monitoring (every 3–6 months, pre-emptive strategy) in the absence of antiviral prophylaxis. 89,8% of pOBI patients were on treatment with anti-CD20 (ocrelizumab or rituximab). Notably, pre-emptive strategy was adopted in all patients with HBsAb > 100 mIU/mL. Conversely, 19 patients neither monitored HBV-DNA nor received antiviral prophylaxis. In detail, 53% of patients with negative or < 100 mIU/mL HBsAb and 30% of patients with HBsAb > 100 mIU/mL did not receive neither prophylaxis nor monitoring. Antiviral prophylaxis was more often prescribed in patients with HBsAb < 100 mIU/mL. Nonetheless, no significant association was found between the choice of HBV reactivation prevention strategy and HBsAb titres < or > than 100 mIU/mL. However, no cases of HBV reactivation were detected in both groups.

## Discussion

In this multicentric study, we showed that patients affected by MS treated with B cells depleting DMTs are very likely to receive complete HBV screening before commencing treatment in clinical practice. Nonetheless, more than half of patients older than 50 years reported no HBV vaccination or had vaccination history missing, and a high proportion of them were HBsAb negative or with a titer lower than 100 UI/mL. Thus, vaccination coverage is still lacking in a large part of Italian MS population, increasing the risk of de-novo severe forms of HBV infection, considering the exposure to immunodepleting DMTs. In particular, 24% of pOBI patients resulted to have higher risk of HBV reactivation, because of negative or < 100 mIU/mL HBsAb titer. Even though no definite recommendations exist for the choice of the best HBV reactivation prevention in such a population, we observed a trend to prescribing antiviral prophylaxis in patients with negative or < 100 mIU/mL HBsAb more often than in patients with HBsAb > 100 mIU/mL, even if the difference was not statistically significant.

No HBV reactivation was reported over 15 months of follow-up, inthis small number of pOBI patients. It is noteworthy, however, from previous studies including patients receiving monoclonal antibodies for rheumatic diseases, that pOBI patients with negative HBsAb are at higher risk of reactivation, while HBsAb > 100 mIU/mL could be considered a protective factor for HBV reactivation [8, 17].

Thus, considering the results of our study, antiviral prophylaxis could be prescribed in MS patients receiving B-cell depleting agents with pOBI and HBsAb negative or < 100 mIU/mL, while in pOBI patients with HBsAb > 100 mIU/mL HBV DNA monitoring without prophylaxis could be a valid and cost-effective measure. However, further studies are required to confirm these observational findings. Nonetheless, the fact that 35% of pOBI patients failed to receive both preventive measures (prophylaxis and monitoring), highlights the urgency of implementing a jointed protocol for HBV reactivation prevention among MS Centers, with a collaboration between neurologists and infectious diseases’ specialists.

Together with the screening and prophylaxis approach, vaccination remains one of the cornerstones in HBV prevention. Among MS patients enrolled in our cohort, a trend towards reduction of HBsAb titres was observed in patients receiving ocrelizumab and in those who got vaccinated before starting the DMT, compared to those who were vaccinated after the start of the therapy, although the difference was not statistically significant. Since data on vaccination schedules and on dates of administration and HBsAb titres determination were too scarce, this difference can be imputable to different timing of HBsAb determination in the two groups.

Nonetheless, a reduction in HBsAb titres even below protective level is expected in patients receiving B-cell depleting agents, thus requiring frequent HBsAb monitoring in pOBI patients with initial titres > 100 mIU/mL, especially when HBV reactivation prevention is managed with pre-emptive strategy. The opportunity to start antiviral prophylaxis, indeed, should be reconsidered on the basis of the point by point HBsAb determination, rather than baseline HBsAb [8, 17].

Although Italy has one of the highest prevalence of HBV infection in Europe, despite its reduction following compulsory HBV vaccination for all the newborns after 1991, we still do not know the prevalence of MS patients with chronic HBV infection or pOBI in real life since no studies have ever been conducted so far. Indeed, in the majority of clinical trials on non anti-CD20 DMTs, screening for HBV was not performed or was only focused on HBsAg, while in phase 3 trials of ocrelizumab, pOBI patients were screened and monitored with HBV DNA every 12 weeks while on treatment, but the proportion of such patients, as well as the proportion of virological or clinical reactivations, were not reported by the authors [18–20]. The results of this study, even if derived from an Italian real-life cohort, could be generalized for other countries, even if we expect to find higher or lower percentage of pOBI and chronically infected patients depending of historical HBV prevalence (during the previous 30–40 years) according to the geographic areas (higher in Mediterranean area and Asia, lower in North America and North Europe), and to vaccination coverages. Thus, the urge of pOBI identification and prophylaxis could be variable according to these considerations.

## Conclusions

To the best of our knowledge, this is the first study evaluating HBV serostatus, vaccination and prophylaxis in a population of MS patients receiving B-cell suppressant DMTs The main limitation of this study is the retrospective design, the scarcity of data about HBsAb titres and the incomplete follow-up. Further prospective studies are needed to define the best follow-up strategy for pOBI MS patients. From this study, we can conclude that a HBV vaccination campaign is needed to target our largely unvaccinated MS population. In particular, we hope that the results of this study will raise the awareness to actively vaccinate all patients diagnosed with MS, considering the possible necessity of using immunosuppressive DMTs in their disease course over time. In the next future, our study group is considering an active vaccination strategy for HBV in MS patients, and will evaluate prospectively the effectiveness of such campaign and its efficacy in long term in MS patients treated with DMTs.
