# Correction to: Management of hepatitis B virus prophylaxis in patients treated with disease-modifying therapies for multiple sclerosis: a multicentric Italian retrospective study

**DOI:** 10.1007/s00415-022-11066-2

**Published:** 2022-04-04

**Authors:** Antonio Riccardo Buonomo, Giulio Viceconte, Massimiliano Calabrese, Giovanna De Luca, Valentina Tomassini, Paola Cavalla, Giorgia Teresa Maniscalco, Diana Ferraro, Viviana Nociti, Marta Radaelli, Maria Chiara Buscarinu, Damiano Paolicelli, Alberto Gajofatto, Pietro Annovazzi, Federica Pinardi, Massimiliano Di Filippo, Cinzia Cordioli, Emanuela Zappulo, Riccardo Scotto, Ivan Gentile, Antonio Luca Spiezia, Martina Petruzzo, Marcello De Angelis, Vincenzo Brescia Morra, Claudio Solaro, Claudio Gasperini, Eleonora Cocco, Marcello Moccia, Roberta Lanzillo, Raising Italian Researchers in Multiple Sclerosis (RIREMS) study group

**Affiliations:** 1 Department of Clinical Medicine and Surgery—Section of Infectious Diseases, University of Naples “Federico II”, Via Sergio Pansini 5, 80130 Naples, Italy; 2 Department of Neuroscience, Biomedicine and Movement, The Multiple Sclerosis Center of the University Hospital of Verona, Verona, Italy; 3 Institute for Advanced Biomedical Technologies (ITAB), Department of Neurosciences, Imaging and Clinical Sciences, University “G. d’Annunzio” of Chieti-Pescara, Chieti, Italy; 4 Department of Neuroscience and Mental Health-City of Health and Science University, Hospital of Torino, Multiple Sclerosis Center, Turin, Italy; 5 Multiple Sclerosis Center, “A. Cardarelli” Hospital, Naples, Italy; 6 Department of Biomedical, Metabolic and Neurosciences, University of Modena and Reggio Emilia, Modena, Italy; 7 Institute of Neurology, Fondazione Policlinico Universitario ‘A. Gemelli’ IRCCS, Catholic University of Sacred Heart, Rome, Italy; 8 Department of Neurology and Multiple Sclerosis Center, ASST Papa Giovanni XXIII, Bergamo, Italy; 9 Department of Neuroscience, Mental Health and Sensory Organs, Sapienza University, S. Andrea Hospital Site, Rome, Italy; 10 Department of Basic Medical Sciences, Neuroscience and Sense Organs, University of Bari “Aldo Moro”, Bari, Italy; 11 Multiple Sclerosis Center, ASST Valle Olona–Gallarate Hospital, Gallarate, VA Italy; 12 UOSI Multiple Sclerosis Rehabilitation, IRCCS, Bologna, Italy; 13 Section of Neurology, Department of Medicine and Surgery, University of Perugia, Perugia, Italy; 14 Multiple Sclerosis Center, Montichiari Hospital, ASST Spedali Civili Di Brescia, Brescia, Italy; 15 Department of Neurosciences, Reproductive and Odontostomatological Sciences, University of Naples “Federico II”, Naples, Italy; 16 CRRF Mons. Luigi Novarese, Moncrivello, VC Italy; 17 Department of Neuroscience, San-Camillo Forlanini Hospital, Rome, Italy; 18 Multiple Sclerosis Center, Binaghi Hospital, Azienda Tutela Della Salute (ATS) Sardegna, Cagliari, Italy

## Correction to: Journal of Neurology 10.1007/s00415-022-11009-x

The original version of this article unfortunately contained a mistake. The given and family names of authors were Interchanged and Dr. Spiezia has two given names (Antonio Luca).

The author names should be

Antonio Riccardo Buonomo, Giulio Viceconte, Massimiliano Calabrese, Giovanna De Luca, Valentina Tomassini, Paola Cavalla, Giorgia Teresa Maniscalco, Diana Ferraro, Viviana Nociti, Marta Radaelli, Maria Chiara Buscarinu, Damiano Paolicelli, Alberto Gajofatto, Pietro Annovazzi, Federica Pinardi, Massimiliano Di Filippo, Cinzia Cordioli, Emanuela Zappulo, Riccardo Scotto, Ivan Gentile, Antonio Luca Spiezia, Martina Petruzzo, Marcello De Angelis, Vincenzo Brescia Morra, Claudio Solaro, Claudio Gasperini, Eleonora Cocco, Marcello Moccia, Roberta Lanzillo

The original article has been corrected.

